# Intermittent Fasting Targets Osteocyte Neuropeptide Y to Relieve Osteoarthritis

**DOI:** 10.1002/advs.202400196

**Published:** 2024-07-08

**Authors:** Yu‐Xuan Qian, Shan‐Shan Rao, Yi‐Juan Tan, Zun Wang, Hao Yin, Teng‐Fei Wan, Ze‐Hui He, Xin Wang, Chun‐Gu Hong, Hai‐Jin Zeng, Yi Luo, Yan‐Xin Duan, Hao Zhu, Xin‐Yue Hu, Ling Zou, Yan Zhang, Bing‐Bing Liu, Zhen‐Xing Wang, Wei Du, Chun‐Yuan Chen, Hui Xie

**Affiliations:** ^1^ Department of Orthopedics Movement System Injury and Repair Research Center Xiangya Hospital Central South University Changsha Hunan 410008 China; ^2^ Hunan Key Laboratory of Angmedicine Changsha Hunan 410008 China; ^3^ Department of Respiratory Medicine Xiangya Hospital Central South University Changsha Hunan 410008 China; ^4^ Department of Pediatrics Union Hospital Tongji Medical College Huazhong University of Science and Technology Wuhan 430022 China; ^5^ School of Computer Science and Engineering Central South University Changsha Hunan 410083 China; ^6^ Department of Rehabilitation Xiangya Hospital Central South University Changsha Hunan 410008 China; ^7^ National Clinical Research Center for Geriatric Disorders Xiangya Hospital Central South University Changsha Hunan 410008 China

**Keywords:** fasting, neuropeptide Y, osteoarthritis, therapeutics

## Abstract

Osteoarthritis is a highly prevalent progressive joint disease that still requires an optimal therapeutic approach. Intermittent fasting is an attractive dieting strategy for improving health. Here this study shows that intermittent fasting potently relieves medial meniscus (DMM)‐ or natural aging‐induced osteoarthritic phenotypes. Osteocytes, the most abundant bone cells, secrete excess neuropeptide Y (NPY) during osteoarthritis, and this alteration can be altered by intermittent fasting. Both NPY and the NPY‐abundant culture medium of osteocytes (OCY‐CM) from osteoarthritic mice possess pro‐inflammatory, pro‐osteoclastic, and pro‐neurite outgrowth effects, while OCY‐CM from the intermittent fasting‐treated osteoarthritic mice fails to induce significant stimulatory effects on inflammation, osteoclast formation, and neurite outgrowth. Depletion of osteocyte NPY significantly attenuates DMM‐induced osteoarthritis and abolishes the benefits of intermittent fasting on osteoarthritis. This study suggests that osteocyte NPY is a key contributing factor in the pathogenesis of osteoarthritis and intermittent fasting represents a promising nonpharmacological antiosteoarthritis method by targeting osteocyte NPY.

## Graphical abstract

Osteocytes, the most abundant bone cells, secrete excess neuropeptide Y (NPY) to promote the development of osteoarthritis by directly or indirectly stimulating inflammation, osteoclast formation, and sensory nerve outgrowth. Intermittent fasting potently relieves osteoarthritis by suppressing the generation of osteocyte NPY and represents a promising nonpharmacological antiosteoarthritis strategy.

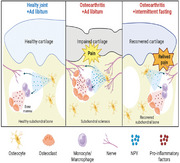

## 1 Introduction

Osteoarthritis is a highly prevalent progressive joint disease characterized by functional impairment, joint pain, cartilage degeneration, synovial inflammation, and subchondral bone structural changes (including sclerosis, osteophyte formation, and cystic lesions).^[^ ^1^ ^]^ This disorder affects over 500 million people worldwide especially the elderly.^[^ ^2^ ^]^ There is currently no ideal medical option for osteoarthritis because the underlying mechanism is not well understood, thus ultimately requiring joint replacement surgery for treatment.^[^ ^3^ ^]^

Intermittent fasting refers to an eating pattern that alternates between periods of fasting (typically ≥12 h) and normally consuming food during the re‐feeding period, which is a very attractive dieting strategy for improving health.^[^ ^4^ ^]^ Intermittent fasting has been shown to exert various health benefits, such as reducing body weight, modulating body composition, preventing cancer, downregulating inflammation, attenuating oxidative stress, improving insulin resistance, enhancing nerve regeneration and longevity, and promoting wound healing.^[^ ^4^, ^5^, ^6^, ^7^ ^]^ Previously published clinical data have revealed that medically supervised fasting for 8 consecutive days (≈300 kcal day^−1^) can reduce pain and improve joint function in individuals with osteoarthritis.^[^ ^8^, ^9^ ^]^ An animal study by Park et al. has shown that intermittent fasting combined with a high‐protein diet can mitigate osteoarthritic symptoms in ovariectomized rats with Alzheimer's disease‐like dementia and osteoarthritis.^[^ ^10^ ^]^ These studies indicate that fasting might be a promising nonpharmacological anti‐osteoarthritis strategy. However, more comprehensive and in‐depth studies are still required to fully understand the effects of intermittent fasting alone on osteoarthritis. Moreover, although the researchers hypothesized that the protective effects of fasting on osteoarthritis may be associated with the increase of lean body mass and decrease of inflammation,^[^ ^10^ ^]^ there is a lack of information regarding the detailed molecular mechanism of intermittent fasting on osteoarthritis.

Neuropeptide Y (NPY) is a highly conserved peptide (36‐amino acid) abundantly expressed in the brain, especially in the hypothalamus.^[^ ^11^ ^]^ In the peripheral organs, NPY is mainly detected in the bone tissues and produced primarily by osteocytes and then by osteoblasts.^[^ ^12^, ^13^ ^]^ NPY regulates multiple biological processes such as nerve growth and regeneration, appetite, digestion, stress response, cardiovascular function, immune activity, inflammation, tumor growth, and bone metabolism.^[^ ^13^, ^14^, ^15^, ^16^ ^]^ Recently, we have found that NPY expression is increased in osteocytes during aging or estrogen deficiency, and excess osteocyte NPY production leads to bone‐fat imbalance and osteoporosis.^[^ ^13^ ^]^ Studies have shown that the concentration of NPY in synovial fluid and cartilage of the knee is much higher in patients with osteoarthritis than in healthy controls.^[^ ^17^ ^]^ Intra‐articular injection of NPY can aggravate chondrocyte hypertrophy and cartilage degradation in mice with knee osteoarthritis,^[^ ^18^ ^]^ suggesting that NPY localized in the joints promotes the progression of osteoarthritis. Subchondral bone offers mechanical support for articular cartilage and its abnormal remodeling contributes importantly to the onset and development of osteoarthritis.^[^ ^19^ ^]^ Currently, it is still unclear whether osteocytes, the most abundant (90–95%) bone cells within the bone matrix^[^ ^20^ ^]^ and one of the most abundant sources of NPY in the periphery,^[^ ^12^, ^13^ ^]^ are involved in the pathogenesis of osteoarthritis and the protective effects of intermittent fasting against osteoarthritis through NPY. Fasting has been shown to facilitate NPY expression in the hypothalamus,^[^ ^21^ ^]^ but notably reduces serum NPY concentration.^[^ ^22^ ^]^ These findings inspired us to explore whether the suppression of osteocyte NPY production is a critical mechanism by which intermittent fasting induces benefits on osteoarthritis.

Here, we generated two experimental osteoarthritis models in mice induced by surgical destabilization of the medial meniscus (DMM) and natural aging, respectively. Then, we comprehensively evaluated the therapeutic effects of intermittent fasting against osteoarthritis in these mice, including physical function, pain sensitivity, articular cartilage structure, subchondral bone remodeling, osteoclast formation, inflammatory activity, and sensory innervation. Next, we assessed whether osteocyte NPY production was augmented during osteoarthritis and whether this alteration could be altered by intermittent fasting. Furthermore, we determined the pro‐inflammatory, pro‐osteoclastic, and pro‐neurite outgrowth effects of osteocyte NPY, and evaluated whether NPY deletion in osteocytes could attenuate DMM‐induced osteoarthritic phenotypes and affect the antiosteoarthritic effects of intermittent fasting. Our study aimed to provide detailed evidence for the protective benefits of intermittent fasting on osteoarthritis and uncover the role of osteocyte NPY during this process.

## 2 Result

### 2.1 Intermittent Fasting Improves Physical Function and Decreases Pain Sensitivity in DMM Mice

**Figure 1:**
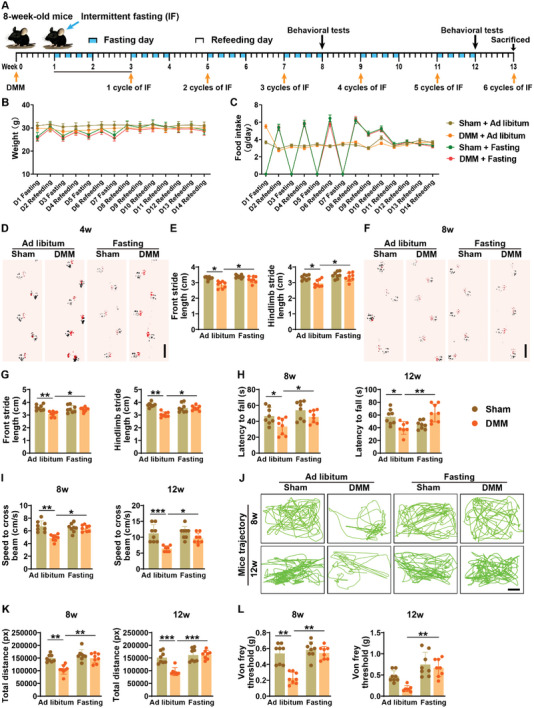
Intermittent fasting improves physical function and decreases pain sensitivity in DMM‐induced osteoarthritic mice. A) Schematic diagram of the experimental design for evaluating the protective effects of different cycles (1, 2, 4, and 6 cycles) of intermittent fasting on DMM‐induced osteoarthritis. B,C) Body weight B) and food consumption C) of sham and DMM mice receiving ad libitum feeding or intermittent fasting in two weeks of the observation period. *n* = 8 per group. D–G) Representative images of footprint patterns and quantification of the stride lengths of forelimb (FL) and hindlimb (HL) in sham and DMM mice with ad libitum feeding or intermittent fasting for 4 D,E) or 8 F,G) weeks assessed by footprint test. Scale bar: 2 cm. *n* = 8 per group. H,I) Latency to fall from the rotarod and speed to cross the balance beam were assessed by the rotarod test H) and balance beam test I) in mice with different treatments for 8 or 12 weeks. *n* = 8 per group. J,K) The locomotor activity of mice receiving different treatments for 8 or 12 weeks was evaluated by 1‐h video motor tracking. The representative motion trajectory diagram was shown in J) and the total movement distance was displayed in K). Scale bar: 10 cm. *n* = 8 per group. L) Pain sensitivity was examined by the paw withdrawal threshold test. *n* = 8 per group. Data are presented as mean ± SD. Two‐way ANOVA combined with Bonferroni *post hoc* test. ^*^ *p* < 0.05, ^**^ *p* < 0.01, ^***^ *p* < 0.001.

To determine the therapeutic effects of intermittent fasting against osteoarthritis, we generated a mouse model of posttraumatic knee osteoarthritis induced by DMM surgery (**Figure** **1**A), which mimics meniscal injury‐induced osteoarthritis in humans. After surgery for 1 week, these mice or the sham‐operated mice received one, two, four, or six cycles of intermittent fasting (alternate‐day fasting for 1 week and then refeeding for another week) or ad libitum feeding (Figure 1A). As shown in Figure 1B,C, intermittent fasting just caused a remarkable reduction of body weight values in both sham‐ and DMM‐operated mice on fasting days, but this alteration was profoundly reversed on refeeding days, which were likely due to the marked increase of food consumption in these mice. During the refeeding week, the appetite of these intermittent fasting‐treated sham and DMM mice gradually returned to a similar level as before and their body weights were comparable to that of sham and DMM mice without receiving fasting (Figure 1B,C). Footprint test indicated that the DMM‐treated mice exhibited significantly decreased forelimb and hindlimb stride lengths compared with the ad libitum‐fed mice, whereas intermittent fasting for 4 or 8 weeks (two or four cycles of intermittent fasting) markedly suppressed the reduction of these parameters (Figure 1D–G), indicating that intermittent fasting improves gait performance in osteoarthritic mice. Rotarod test and balance beam test, respectively, revealed that the DMM mice showed prominent decreases in the latency to fall from the rotarod and the speed to cross the balance beam, but these changes were significantly abolished in DMM mice receiving intermittent fasting for 8 weeks (Figure 1H,I). One‐hour video tracking for activity analysis showed that the DMM‐induced movement impairment could be notably reversed with 8 or 12 weeks of intermittent fasting, to the extent observed in the sham‐operated control mice (Figure 1J,K). The paw withdrawal threshold test indicated a much higher pain sensitivity in DMM mice compared with the sham mice, but not in the DMM‐treated mice receiving intermittent fasting for 8 or 12 weeks (Figure 1L). A trend of increase in Von Frey threshold was also observed in sham mice treated with six cycles of intermittent fasting (12 weeks; Figure 1L), suggesting the reduction of pain sensitivity of these normal control mice in response to intermittent fasting. These results indicate that intermittent fasting can efficiently improve physical function and attenuate pain hypersensitivity in DMM‐induced osteoarthritic mice.

### 2.2 Intermittent Fasting Stabilizes Subchondral Bone Microarchitecture and Attenuates Cartilage Degeneration in DMM Mice

**Figure 2:**
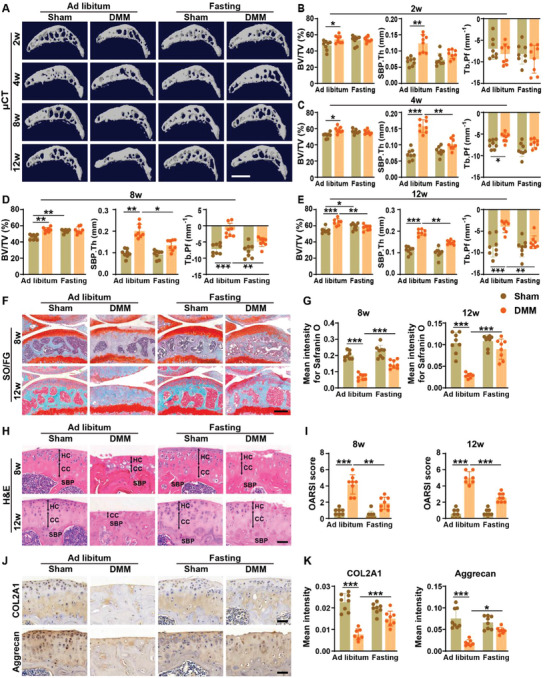
Intermittent fasting stabilizes subchondral bone microarchitecture and attenuates cartilage degeneration in DMM mice. A–D) Representative µCT scanning images A) and quantitative analysis of subchondral bone volume fraction (Tb. BV/TV), subchondral bone plate thickness (SBP. Th), and trabecular pattern factor (Tb. Pf) in the tibial subchondral bones from mice receiving ad libitum feeding or intermittent fasting for 2 B), 4 C), 8 D), or 12 E) weeks. Scale bars: 500 µm. *n* =8 per group. F,G) Representative Safranin O‐fast green staining images F) and quantification of the mean intensity of the Safranin O‐stained articular cartilages G) from mice receiving ad libitum feeding or intermittent fasting for 8 or 12 weeks. Scale bars: 250 µm. *n* = 8 per group. H,I) Representative HE staining images H) and quantification of OARSI scores I) in the articular cartilages from mice with different treatments for 8 or 12 weeks. HC: hyaline cartilage; CC: calcified cartilage; SBP: subchondral bone plate. Scale bars: 50 µm. *n* = 8 per group. J,K) Representative COL2A1 and aggrecan staining images J) and quantitative analysis of the mean intensity of the COL2A1‐or aggrecan‐positive articular cartilages K) from mice with different treatments for 12 weeks. Scale bars: 50 µm. *n* = 8 per group. Data are presented as mean ± SD. Two‐way ANOVA combined with Bonferroni *post hoc* test. ^*^ *p* < 0.05, ^**^ *p* < 0.01, ^***^ *p* < 0.001.

**Figure** **2**A–D shows the microcomputed tomography (µCT) scanning images of the tibial subchondral bones and the quantitative data of subchondral bone volume fraction (Tb. BV/TV), subchondral bone plate thickness (SBP. Th), and trabecular pattern factor (Tb. Pf). The results showed that Tb. BV/TV, SBP. Th, and/or Tb. Pf were markedly increased at different time points after DMM surgery, whereas these changes were significantly blocked in DMM + intermittent fasting mice (Figure 2A–E), which indicated that intermittent fasting mitigated DMM‐induced aberrant subchondral bone remodeling and improved subchondral bone microarchitecture. Safranin O‐fast green staining revealed the loss of cartilage glycosaminoglycans and proteoglycan in the tibial cartilage of the DMM mice at 9 weeks after surgery (8 weeks after ad libitum feeding), which was further exacerbated at the later time point (Figure 2F,G). Aberrant bone formation was observed in the tibial subchondral bone of the DMM mice at 12 weeks after treatment, but this change was not as significant in DMM + intermittent fasting mice (Figure 2F). Consistently, osteocalcin (OCN) immunostaining showed that the DMM + Ad libitum mice exhibited a significant increase of osteoblast number in tibial subchondral bone at 2, 4, 8, and 12 weeks after treatment (Figure S1A,B, Supporting Information), suggesting that DMM surgery markedly stimulates osteogenesis. However, although intermittent fasting could also promote osteogenesis in the sham‐operated mice, the DMM surgery did not induce a further increase of osteoblast number in the intermittent fasting‐treated mice (Figure S1A,B, Supporting Information), suggesting that intermittent fasting can block DMM‐induced aberrant osteogenesis. Hematoxylin and eosin (H&E) staining showed that the thickness of the hyaline cartilage (HC) zone was profoundly decreased in DMM mice receiving ad libitum feeding for 8 weeks and the hyaline cartilage loss progressed even further, with the calcified cartilage (CC) zone moving to the articular surface at 12 weeks (Figure 2H). However, glycosaminoglycans and proteoglycan loss were profoundly inhibited and a large area of hyaline cartilage was maintained in DMM + intermittent fasting mice (Figure 2F–H). The protective effects of intermittent fasting against the DMM‐induced articular cartilage degeneration were confirmed by the much lower Osteoarthritis Research Society International (OARSI) score (Figure 2I) and lower mean staining intensities for two major components of cartilage extracellular matrix (COL2A1 and aggrecan) at 8 weeks after treatment (Figure 2J,K). These results reveal that intermittent fasting can stabilize the subchondral bone and alleviate cartilage degeneration in DMM‐induced osteoarthritic mice.

### 2.3 Intermittent Fasting Suppresses Osteoclast Formation, Inflammatory Activity, and Sprouting of CGRP‐Positive Sensory Nerves in DMM Mice

**Figure 3:**
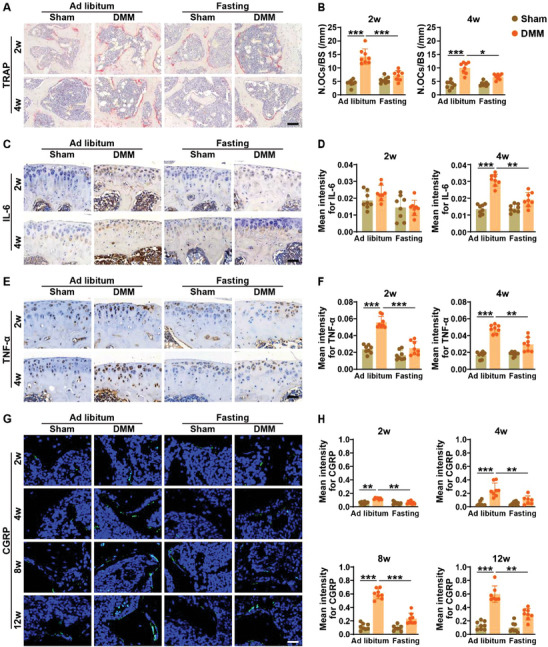
Intermittent fasting suppresses osteoclast formation, inflammation, and sprouting of CGRP‐positive sensory nerves in DMM mice. A,B) Representative TRAP staining images A) and quantification of the numbers of the TRAP‐stained osteoclasts per bone surface B; N. OCs/BS/mm) in the tibial subchondral bones from mice receiving ad libitum feeding or intermittent fasting for 2 or 4 weeks. Scale bars: 100 µm. *n* = 8 per group. C,D) Representative images of immunohistochemical staining for IL‐6 C) and quantification of the mean intensity of the IL6‐positive areas D) in the tibial cartilages and subchondral bone marrow from mice with different treatments for 2 or 4 weeks. Scale bars: 50 µm. *n* = 8 per group. E,F) Representative TNF‐α staining images E) and quantification of the mean intensity of the TNF‐α‐positive areas F) in the tibial cartilages and subchondral bone marrow. Scale bars: 50 µm. *n* = 8 per group. G,H) Representative CGRP staining images G) and quantification of the mean intensity of the CGRP‐stained sensory nerve fibers H) in subchondral bone marrow from mice with different treatments for 2, 4, 8, or 12 weeks. Scale bars: 50 µm. *n* = 8 per group. Data are presented as mean ± SD. Two‐way ANOVA combined with Bonferroni *post hoc* test. ^*^ *p* < 0.05, ^**^ *p* < 0.01, ^***^ *p* < 0.001.

We next assessed the changes in osteoclast number in sham and DMM mice with or without fasting treatment. As evidenced by tartrate‐resistant acid phosphatase (TRAP) staining, the number of osteoclasts was prominently enhanced in the tibial subchondral bone after DMM, as compared with the sham‐operated mice (**Figure** **3**A,B). One cycle (2 weeks) of intermittent fasting was sufficient to reduce osteoclast number in DMM mice, and the suppression of osteoclast formation became much more notable after another cycle of intermittent fasting (Figure 3A,B). Immunohistochemical staining for interleukin 6 (IL‐6) and tumor necrosis factor‐α (TNF‐α) showed much higher levels of these pro‐inflammatory factors in DMM mice, but the alterations were remarkably suppressed in DMM + intermittent fasting mice (Figure 3C–F). The increase of calcitonin gene‐related peptide (CGRP)‐positive sensory nerve innervation within bone is associated with joint pain during osteoarthritis.^[^ ^23^ ^]^ Figure 3G,H shows a notable increase in the density of the CGRP‐immunoreactive sensory nerve fibers in subchondral bone marrow as early as 3 weeks after DMM (2 weeks after ad libitum feeding), and the continued upregulation was detected at the following time points (Figure 3G,H), consistent with the evidence showing pain hypersensitivity in DMM mice. However, the accumulation of these sensory nerves was significantly inhibited with intermittent fasting treatment (Figures 3G,H), consistent with the decrease of pain sensitivity in DMM + intermittent fasting mice. These results indicate that treatment with intermittent fasting from the early stage of osteoarthritis can suppress osteoclast formation, inflammatory activity, and sprouting of CGRP‐positive sensory nerves.

### 2.4 Intermittent Fasting Mitigates Aging‐Induced Osteoarthritic Phenotypes

**Figure 4:**
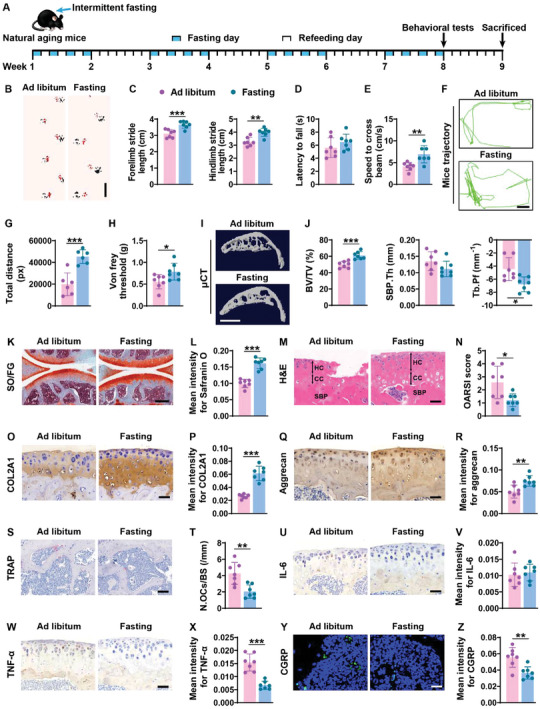
Intermittent fasting mitigates aging‐induced osteoarthritic phenotypes. A) Schematic diagram of the experimental design for evaluating the therapeutic effects of 8 weeks (four cycles) of intermittent fasting against aging‐induced osteoarthritis in 16‐month‐old mice. B,C) Representative images of footprint patterns B) and quantification of forelimb and hindlimb stride lengths C). Scale bar: 2 cm. *n* = 7 per group. D,E) Latency to fall from the rotarod D) and speed to cross the balance beam E). *n* = 7 per group. F,G) Representative motion trajectory diagram F) and quantification of total movement distance G). *n* = 7 per group. H) Pain sensitivity measured by paw withdrawal threshold test. *n* = 7 per group. I,J) Representative µCT scanning images of the tibial subchondral bones I) and quantification of Tb. BV/TV, SBP. Th, and Tb. Pf J). Scale bar: 1 mm. *n* = 7 per group. K,L) Representative Safranin O‐fast green staining images K) and quantification of the mean intensity for the Safranin O‐positive tibial cartilages L). Scale bars: 250 µm. *n* = 7 per group. M,N) Representative HE staining images M) and quantification of OARSI scores N). Scale bars: 50 µm. *n* = 7 per group. O–R) Representative staining images and quantification of the mean intensities for COL2A1 O,P) and aggrecan Q,R). Scale bars: 50 µm. *n* = 7 per group. S,T) Representative TRAP staining images S) and quantification of osteoclast number T). Scale bars: 100 µm. *n* = 7 per group. U–Z) Representative staining images and quantification of the mean intensities for IL‐6 U,V; Scale bars: 50 µm), TNF‐α W,X; Scale bars: 50 µm), and CGRP Y,Z; Scale bars: 50 µm). *n* = 7 per group. Data are presented as mean ± SD. Unpaired, two‐tailed student's *t*‐test. ^*^ *p* < 0.05, ^**^ *p* < 0.01, ^***^ *p* < 0.001.

Aging is the major risk factor for primary osteoarthritis.^[^ ^24^ ^]^ We then evaluated whether intermittent fasting for two cycles (8 weeks) can induce therapeutic benefits in 16‐month‐old mice (**Figure** **4**A). Consistent with that observed in the DMM‐induced osteoarthritic mice, behavioral tests including the footprint test, the rotarod test, the balance beam test, and movement tracking revealed that intermittent fasting treatment resulted in the longer forelimb and hindlimb stride lengths (Figure 4B,C), a trend of increase in latency to fall from the iron rod (Figure 4D), a faster speed to pass through the balance beam (Figure 4E), and a higher extent of locomotor activity (Figure 4F,G) relative to the control mice, indicating that intermittent fasting successfully improves physical function in aged mice. Moreover, the intermittent fasting‐treated aged mice showed a much higher paw withdrawal threshold compared to the control mice (Figure 4H), indicating an increase in pain tolerance in aged mice after intermittent fasting treatment.

Subsequently, we compared the structures of subchondral bone and articular cartilage between the control and intermittent fasting‐treated aged mice. As indicated by µCT analysis, intermittent fasting increased subchondral Tb. BV/TV in these aged mice, but caused a significant reduction of Tb. Pf and a trend of decrease in SBP. Th (Figure 4I,J). Safranin O‐fast green staining and H&E staining revealed a much lower degree of cartilage degenerative phenotypes in response to intermittent fasting, as shown by an increased area of Safranin O‐stained articular cartilage (Figure 4K,L), a thicker hyaline cartilage zone (Figure 4M), a lower value of OARSI score (Figure 4N), and higher mean staining intensities for COL2A1 and aggrecan (Figure 4O–R) in the intermittent fasting‐treated aged mice relative to the control mice. Thus, intermittent fasting can also improve subchondral bone microarchitecture and prevent articular cartilage degeneration in aged mice.

Consistent with the above results showing the improved subchondral bone structure, attenuated cartilage degeneration, and decreased pain sensitivity, treatment of aged mice with intermittent fasting dramatically reduced the number of subchondral bone osteoclasts (Figure 4S,T), the expression of pro‐inflammatory factors (Figure 4U–X), and the density of the CGRP‐positive sensory nerve fibers (Figure 4Y,Z) relative to the controls, as revealed by TRAP staining, immunohistochemical staining for IL‐6 and TNF‐α, and immunofluorescence staining for CGRP in tibial subchondral bone and cartilage. These results suggest that the suppression of osteoclast formation, inflammation, and sensory nerve innervation contributes to the protective effects of intermittent fasting against aging‐induced osteoarthritis.

### 2.5 Intermittent Fasting Blocks the DMM‐Induced Increase of the Pro‐Inflammatory, Pro‐osteoclastic, and Pro‐neurite Outgrowth Factor NPY by Osteocytes

**Figure 5:**
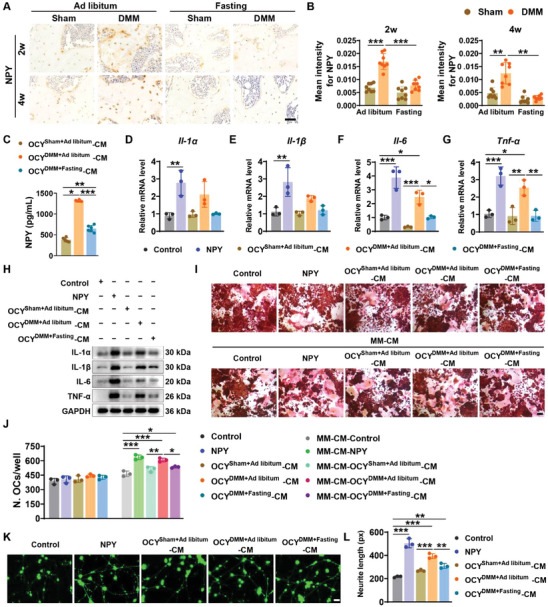
Intermittent fasting blocks the DMM‐induced increase of the pro‐inflammatory, pro‐osteoclastic, and pro‐neurite outgrowth factor NPY by osteocytes. A,B) Representative immunohistochemical staining images of NPY A) and quantification of the mean staining intensity for NPY B) in the tibial subchondral bones from sham‐ or DMM‐operated 3‐month‐old young mice receiving ad libitum feeding or intermittent fasting for 2 or 4 weeks. Scale bars: 50 µm. *n* = 8 per group. C) The concentration of NPY in the culture medium of osteocytes (OCY^Sham^‐CM, OCY^DMM^‐CM, and OCY^DMM + Fasting^‐CM) from sham, DMM, and DMM + intermittent fasting mice assessed by ELISA. *n* = 6 per group. D–G) qRT‐PCR of *Il‐1α* D), *Il‐1β* E), *Il‐6* F), and *Tnf‐α* G) in monocyte/macrophage cell line RAW264.7 with different treatments. *n* = 3 per group. H) Western blot analysis for IL‐1α, IL‐1β, IL‐6, and TNF‐α in RAW264.7 with different treatments. I,J) Representative TRAP staining images I) and quantification of the number of osteoclasts (>3 nuclei; J). Scale bar: 100 µm. *n* = 3 per group. K,L) Representative staining images of calcein AM K) and quantification of the neurite lengths L) in the calcein AM‐stained live CAD cells. Scale bar: 50 µm. *n* = 3 per group. Data are presented as mean ± SD. For panel (B): Two‐way ANOVA with Bonferroni *post hoc* test. For panel (C‐G and L): Unpaired, two‐tailed student's *t*‐test (differences between control and NPY groups, or control and OCY‐CM from different groups) or one‐way ANOVA with Bonferroni *post hoc* test (differences among OCY‐CM or MM‐CM from different groups). ^*^ *p* < 0.05, ^**^ *p* < 0.01, ^***^ *p* < 0.001.

To examine the involvement of NPY in the development of osteoarthritis and the antiosteoarthritic effects of intermittent fasting, we first tested the expression changes of NPY in DMM‐induced osteoarthritis mouse models. Immunohistochemical staining for bone NPY showed that this peptide was located in cells at the bone surface and cells embedded in the bone matrix of the tibial subchondral bone (**Figure** **5**A,B), in accordance with our previous evidence that NPY is mainly expressed by osteoblasts and osteocytes in the bone tissues.^[^ ^13^ ^]^ Once subjected to DMM surgery, the expression of bone NPY especially that in the osteocytes was significantly enhanced, but this change was profoundly reversed by intermittent fasting (Figure 5A,B), indicating that intermittent fasting can reduce osteocyte NPY overproduction in DMM mice. In the brain, a tissue that abundantly expresses NPY, however, showed no significant change of NPY expression upon DMM surgery but exhibited a marked increase of NPY in DMM mice after intermittent fasting treatment (Figure S2A,B, Supporting Information), suggesting that brain NPY is not involved in the pathogenesis of DMM‐induced osteoarthritis and does not contribute to NPY accumulation in the osteoarthritic joint.

We next isolated osteocytes from sham, DMM, and DMM + intermittent fasting mice and assessed the levels of NPY in the culture supernatants from osteocytes in these mice. Enzyme‐linked immunosorbent assay (ELISA) revealed that the culture medium of osteocytes from DMM + Ad libitum mice (OCY^DMM + Ad libitum^‐CM) had a much higher level of NPY compared with those from the sham‐operated mice (OCY^Sham + Ad libitum^‐CM), whereas the peptide level of NPY in OCY‐CM from DMM + intermittent fasting mice (OCY^DMM + Fasting^‐CM) was profoundly decreased relative to OCY^DMM + Ad libitum^‐CM (Figure 5C), in accordance with the changes of bone NPY expression assessed by immunohistochemical staining.

We then explored the effects of culture supernatants from osteocytes in these mice on inflammatory factor expression. Quantitative real‐time polymerase chain reaction (qRT‐PCR) analysis showed that incubation with NPY or OCY^DMM + Ad libitum^‐CM, but not from OCY^Sham + Ad libitum^‐CM, markedly increased the mRNA levels of *Il‐1α*, *Il‐1β*, *Il‐6*, or/and *Tnf‐α* in RAW264.7 (a mouse monocyte/macrophage cell line; Figure 5D–G). However, OCY^DMM + Fasting^‐CM failed to notably increase the expression of these pro‐inflammatory genes (Figure 5D–G). The pro‐inflammatory properties of NPY and OCY^DMM + Ad libitum^‐CM were further determined by western blot analysis of the protein expression of IL‐1α, IL‐1β, IL‐6, and TNF‐α (Figure 5H). As pro‐inflammatory factors can stimulate osteoclastogenesis,^[^ ^25^ ^]^ we collected the culture media from the above‐treated monocytes/macrophages and tested their effects on osteoclast formation. TRAP staining showed that NPY protein, OCY^Sham + Ad libitum^‐CM, OCY^DMM + Ad libitum^‐CM, and OCY^DMM + Fasting^‐CM had no direct significant effects on osteoclast differentiation (Figure 5I,J). However, the culture media from the above OCY^DMM + Ad libitum^‐CM‐ or NPY‐treated monocytes/macrophages (MM‐CM from OCY^DMM + Ad libitum^‐CM and NPY groups), but not MM‐CM from OCY^Sham + Ad libitum^‐CM treatment group, significantly enhanced osteoclast formation of RAW264.7 under osteoclastic induction (Figure 5I,J). The pro‐osteoclastic effect of MM‐CM from OCY^DMM + Fasting^‐CM treatment group was markedly impaired compared with those from OCY^DMM + Ad libitum^‐CM group (Figure 5I,J). CAD cells are a neuronal cell line and the removal of serum from the culture can induce their neuronal differentiation.^[^ ^26^ ^]^ As shown in Figure 5K,L, CAD cells cultured in serum‐free culture medium exhibited neurite outgrowth. When incubated with OCY^DMM + Ad libitum^‐CM or NPY, but not OCY^Sham + Ad libitum^‐CM, markedly increased neurite length in CAD cells under serum‐free conditions, whereas this effect was much lower in OCY^DMM + Fasting^‐CM treatment group (Figure 5K,L). These results, combined with the evidence showing the increase of osteocyte NPY production after DMM surgery, suggest that intermittent fasting may act by targeting osteocyte NPY to suppress inflammation, osteoclast overproduction, and excess neurite outgrowth in osteoarthritic mice.

Since NPY has been shown to function through Y1R to regulate inflammatory activity and neurogenesis of macrophages, we assessed the role of Y1R in the NPY‐ and NPY‐abundant OCY^DMM + Ad libitum^‐CM‐induced promotion of macrophage inflammation and neurite outgrowth. We first tested whether the inhibition of Y1R using the Y1R antagonist BIBO3304 could block the pro‐inflammatory effects of NPY‐ and NPY‐abundant OCY^DMM + Ad libitum^‐CM in RAW264.7 cells. qRT‐PCR showed that both NPY and OCY^DMM + Ad libitum^‐CM could significantly stimulate the gene expression of pro‐inflammatory factors including *Il‐1α*, *Il‐1β*, *Il‐6*, and *Tnf‐α* in RAW264.7 cells, whereas these effects were profoundly impaired by BIBO3304 (Figure S3A–D, Supporting Information). Consistently, calcein‐AM staining revealed that BIBO3304 treatment remarkably blocked the positive effects of NPY and OCY^DMM + Ad libitum^‐CM on neurite outgrowth of CAD cells (Figure S3E,F, Supporting Information). These results indicate that NPY and OCY^DMM + Ad libitum^‐CM mainly function through Y1R to stimulate inflammation and neurite outgrowth.

### 2.6 Depletion of Osteocyte NPY Blocks the Intermittent Fasting‐Induced Therapeutic Benefits in Osteoarthritic Mice

**Figure 6:**
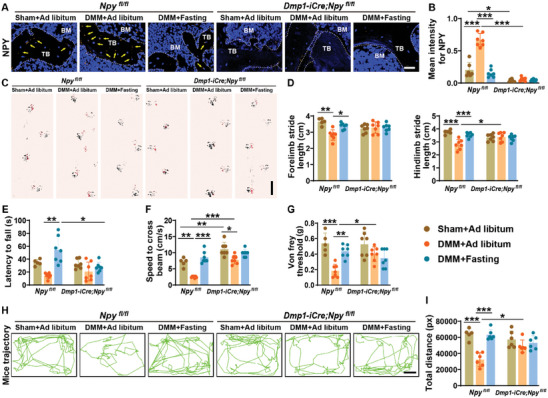
Depletion of osteocyte NPY blocks the intermittent fasting‐induced improvements on physical function and pain sensitivity in osteoarthritic mice. A,B) Representative immunostaining images of NPY A) and quantification of the mean staining intensity for NPY B) in the tibial subchondral bones from sham‐ or DMM‐operated *Npy^fl/fl^* mice and *Dmp1‐iCre*; *Npy^fl/fl^* mice receiving ad libitum feeding or intermittent fasting for 4 weeks. Scale bars: 50 µm. *n* = 6–7 per group. C,D) Representative images of footprint patterns C) and quantification of forelimb and hindlimb stride lengths D) in sham or DMM‐operated *Npy^fl/fl^* mice and *Dmp1‐iCre*; *Npy^fl/fl^* mice receiving ad libitum feeding or intermittent fasting for 8 weeks. Scale bar: 2 cm. *n* = 5–7 per group. E,F) Latency to fall from the rotarod E) and speed to cross the balance beam F). *n* = 5–7 per group. G) Pain sensitivity was assessed by the paw withdrawal threshold test. *n* = 5–7 per group. H,I) Representative motion trajectory diagram H) and quantification of total movement distance I). Scale bar: 10 cm. *n* = 5–7 per group. Data are presented as mean ± SD. Two‐way ANOVA with Bonferroni *post hoc* test. ^*^ *p* < 0.05, ^**^ *p* < 0.01, ^***^ *p* < 0.001.

We previously generated osteocyte NPY knockout (*Dmp1‐iCre*; *Npy^fl/fl^*) mice and reported the contributory role of osteocyte NPY in the development of osteoporosis.^[^ ^13^ ^]^ To determine the role of osteocyte NPY in osteoarthritis, the *Dmp1‐iCre*; *Npy^fl/fl^* mice and their control mice (*Npy^fl/fl^* mice) were subjected to DMM surgery or sham operation. The *Npy^fl/fl^* mice exhibited a notable increase of osteocyte NPY expression in the tibial subchondral bone once receiving DMM and a sharp decline of this peptide was observed after additional treatment with intermittent fasting for 4 weeks (**Figure** **6**A,B). In *Dmp1‐iCre*; *Npy^fl/fl^* mice, osteocyte NPY was rarely detected in sham, DMM, and DMM + intermittent fasting groups (Figure 6A,B). Brain NPY, however, was not notably affected in *Dmp1‐iCre*; *Npy^fl/fl^* mice compared with *Npy^fl/fl^* mice subjected to either sham or DMM surgery, and still significantly increased after intermittent fasting treatment (Figure S4A,B, Supporting Information).

Behavioral tests including the footprint test, rotarod test, balance beam test, and movement tracking showed that treatment with DMM caused notable reductions in the forelimb and hindlimb stride lengths (Figure 6C,D), latency to fall from the rotarod (Figure 6E), speed to cross the balance beam (Figure 6F), Von Frey paw withdrawal threshold (Figure 6G), and total distance of movement (Figure 6H,I) in *Npy^fl/fl^* mice. However, these changes were markedly reversed in DMM‐operated *Npy^fl/fl^* mice receiving intermittent fasting for 8 weeks (Figure 6C–I), in accordance with that observed in wild‐type mice. However, both DMM surgery and intermittent fasting did not induce statistically significant effects on most of these motor function parameters in *Dmp1‐iCre*; *Npy^fl/fl^* mice (Figure 6C–I). A trend of decrease in Von Frey paw withdrawal threshold was just observed in *Dmp1‐iCre*; *Npy^fl/fl^* mice subjected to DMM surgery (Figure 6F).

**Figure 7:**
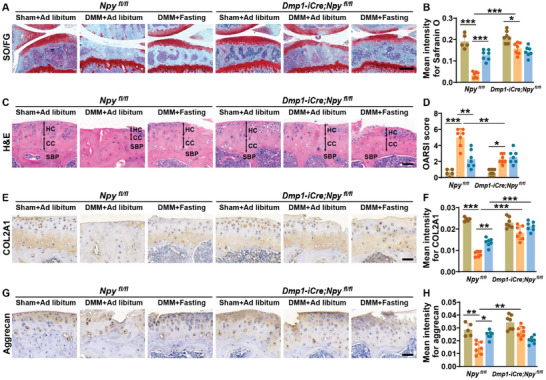
Depletion of osteocyte NPY blocks the intermittent fasting‐induced protective effects against articular cartilage degeneration in osteoarthritic mice. A,B) Representative Safranin O‐fast green staining images A) and quantification of the mean intensity for the Safranin O‐stained tibial cartilages B). Scale bars: 250 µm. *n* = 5–7 per group. C,D) Representative HE staining images C) and quantification of OARSI scores D). Scale bars: 50 µm. *n* = 5–7 per group. E–H) Representative staining images and quantification of the mean staining intensities for COL2A1 E,F) and aggrecan G,H). Scale bars: 50 µm. *n* = 5–7 per group. Data are presented as mean ± SD. Two‐way ANOVA with Bonferroni *post hoc* test. ^*^ *p* < 0.05, ^**^ *p* < 0.01, ^***^ *p* < 0.001.

Safranin O‐fast green and H&E staining of the knee joints showed significant articular cartilage loss and much higher OARSI scores in the tibias of the ad libitum‐fed *Npy^fl/fl^* mice after DMM, but most of the cartilages with large areas of hyaline cartilages were still maintained in the tibias of the DMM + intermittent fasting‐treated *Npy^fl/fl^* mice (**Figure** **7**A–D). DMM also induced a reduction of articular cartilages (mainly the hyaline cartilages) and an increase of OARSI scores in *Dmp1‐iCre*; *Npy^fl/fl^* mice, but the changes were much milder compared with that in *Npy^fl/fl^* mice (Figure 7A–D). Intermittent fasting did not show a protective effect against DMM‐induced articular cartilage loss in *Dmp1‐iCre*; *Npy^fl/fl^* mice, but caused a trend of decrease in Safranin O‐stained cartilages and increase in OARSI scores compared with the ad libitum‐fed *Dmp1‐iCre*; *Npy^fl/fl^* mice (Figure 7A–D), suggesting that intermittent fasting might induce a slightly harmful effect on knee joint in osteocyte NPY‐lacking mice. Immunohistochemical staining for COL2A1 and aggrecan in the tibial articular cartilage showed that DMM significantly reduced the mean staining intensities for these two cartilage matrix components in *Npy^fl/fl^* mice, whereas intermittent fasting effectively blocked these cartilage degenerative changes (Figure 7E–H). However, cartilage degeneration was less notable in DMM + Ad libitum‐treated *Dmp1‐iCre*; *Npy^fl/fl^* mice and there was no improvement in the loss of COL2A1 and aggrecan in the DMM + intermittent fasting‐treated *Dmp1‐iCre*; *Npy^fl/fl^* mice (Figure 7E–H).

**Figure 8:**
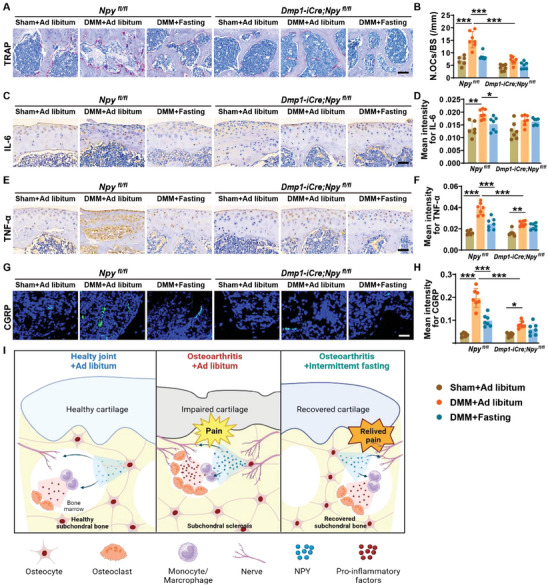
Depletion of osteocyte NPY blocks the intermittent fasting‐induced suppression of osteoclast formation, inflammation, and excess sensory innervation in osteoarthritic mice. A,B) Representative images of TRAP staining A) and quantification of the TRAP‐stained osteoclasts B) in the tibial subchondral bones from sham‐ or DMM‐operated *Npy^fl/fl^* mice and *Dmp1‐iCre*; *Npy^fl/fl^* mice receiving ad libitum feeding or intermittent fasting for 4 weeks. Scale bars: 100 µm. *n* = 5–7 per group. C–F) Representative staining images and quantification of the mean intensities for IL‐6 C,D) and TNF‐α E,F) in the tibial cartilages and subchondral bone marrow from *Npy^fl/fl^* mice and *Dmp1‐iCre*; *Npy^fl/fl^* mice with different treatments for 4 weeks. Scale bars: 50 µm. *n* = 5–7 per group. G,H) Representative images of immunofluorescence staining for CGRP G) and quantification of the mean intensity of the CGRP‐stained sensory nerve fibers H) in subchondral bone marrow from *Npy^fl/fl^* mice and *Dmp1‐iCre*; *Npy^fl/fl^* mice with different treatments for 4 weeks. Scale bars: 50 µm. *n* = 5–7 per group. I) Schematic diagram (created by BioRender) showing the therapeutic effects of intermittent fasting against osteoarthritis through the inhibition of osteocyte NPY. Data are presented as mean ± SD. Two‐way ANOVA with Bonferroni *post hoc* test. ^*^ *p* < 0.05, ^**^ *p* < 0.01, ^***^ *p* < 0.001.

We finally evaluated osteoclast formation, inflammatory responses, and sensory nerve outgrowth in *Dmp1‐iCre*; *Npy^fl/fl^* mice and their control *Npy^fl/fl^* mice receiving sham, DMM, or DMM + intermittent fasting for 4 weeks. As shown by TRAP staining, the DMM‐treated *Npy^fl/fl^* mice displayed a much higher number of subchondral osteoclasts relative to the sham‐operated *Npy^fl/fl^* mice, but the stimulatory effect of DMM on subchondral osteoclast formation was profoundly suppressed in *Dmp1‐iCre*; *Npy^fl/fl^* mice (**Figure** **8**A,B). Intermittent fasting markedly reduced the subchondral osteoclast number in the DMM‐treated *Npy^fl/fl^* mice, while this therapeutic change was not noteworthy in the DMM + intermittent fasting‐treated *Dmp1‐iCre*; *Npy^fl/fl^* mice (Figure 8A,B). Immunostaining for the IL‐6, TNF‐α, and CGRP in the knee joints of the *Npy^fl/fl^* mice indicated that intermittent fasting treatment dramatically attenuated the DMM‐induced increases in pro‐inflammatory factor production (Figure 8C–F) and CGRP‐positive sensory nerve fiber density (Figure 8G,H). In the *Dmp1‐iCre*; *Npy^fl/fl^* mice, the DMM‐induced augmentation of the inflammatory activity and sensory nerve outgrowth was remarkably impaired, and intermittent fasting failed to induce significant inhibitory effects on inflammation and neurite outgrowth (Figure 8C–H).

Collectively, our results indicate an essential role of osteocyte NPY in the development of DMM‐induced osteoarthritic phenotypes (Figure 8I). This protein can directly promote neurite outgrowth and stimulate monocytes/macrophages to produce pro‐inflammatory factors, which enhance the generation of osteoclasts and result in aberrant subchondral bone remodeling (Figure 8I). These findings also indicate that the protective effects of intermittent fasting against osteoarthritis are mediated by inhibiting the production of osteocyte NPY (Figure 8I).

## 3 Discussion

Identifying the biomarkers and therapeutic targets at the initial phase of osteoarthritis is crucial for preventing the disease at an early stage and slowing down its progression. Although the risk factors (aging, gender, obesity, knee injuries, etc.) of osteoarthritis are well characterized, the detailed molecular mechanism that triggers the onset and development of this disease remains elusive.^[^ ^2^ ^]^ Subchondral bone, a bony layer beneath the articular cartilage, provides both mechanical and essential nutrients for the articular cartilage and plays a key role in maintaining its normal structure and function.^[^ ^27^ ^]^ Various microstructural alterations in subchondral bone such as aberrant bone remodeling (early‐phase bone loss and subsequent bone sclerosis) and histopathological changes are involved in the occurrence of cartilage damage and osteoarthritis.^[^ ^19^ ^]^ These alterations are attributed to abnormal interactions among various cell types (e.g., osteocytes, osteoblasts, osteoclasts, and sensory neurons) in the subchondral bone microenvironment.^[^ ^27^ ^]^ At the early stage of osteoarthritis, osteocytes produce excessive receptor activators of nuclear factor κB ligand (RANKL) in response to aberrant mechanical uploading, thus augmenting osteoclast formation and bone resorption.^[^ ^19^, ^27^ ^]^ With progressive cartilage destruction and mechanical overloading in the adjacent subchondral bone, osteocyte sclerostin (SOST) production is inhibited and the secretion of proteins such as Wnt, DMP1, and transforming growth factor‐β1 is enhanced, thus increasing osteoblastic bone formation and leading to subchondral sclerosis.^[^ ^19^, ^28^, ^29^ ^]^ In parallel, osteoblasts secrete a class of factors with pro‐inflammatory, ‐osteoclastic, or ‐nerve sprouting effects, such as IL‐6, metalloproteinases matrix metalloproteinases, vascular endothelial growth factor, and prostaglandin E2.^[^ ^3^, ^19^, ^27^ ^]^ As for osteoclasts, these cells can secrete netrin‐1 to contact CGRP‐positive sensory neurons, resulting in enhanced sensory innervation and osteoarthritic pain.^[^ ^23^ ^]^

In our study, we identified osteocyte NPY as a key regulator that orchestrated most pathological changes during osteoarthritis, including inflammation, robust osteoclast formation, excessive sensory innervation, and cartilage degeneration. Osteocyte NPY could contact with monocytes/macrophages to induce joint inflammation, as incubation with either NPY protein or NPY‐abundant OCY^DMM + Ad libitum^‐CM directly increased multiple pro‐inflammatory cytokine production in monocytes/macrophages and knockout of osteocyte NPY profoundly abolished the stimulation of joint inflammation induced by DMM in mice. Previous evidence also uncovers the pro‐inflammatory role of NPY in macrophages.^[^ ^16^, ^30^ ^]^ Subchondral bone in osteoarthritis undergoes overactivated bone remodeling, in which bone resorption by osteoclasts and bone formation by osteoblasts is enhanced at the early and advanced phases of osteoarthritis, respectively.^[^ ^19^ ^]^ We and others have shown that NPY exerts a negative effect on osteogenesis and has no notable influence on osteoclastogenesis.^[^ ^12^, ^13^ ^]^ Consistently, the results in our study revealed that both NPY or NPY‐abundant OCY^DMM + Ad libitum^‐CM did not induce a marked direct effect on osteoclast differentiation. However, osteocyte NPY depletion notably reduced osteoclast number in osteoarthritic mice. This might be due to the repression of inflammation in osteocyte NPY‐lacking mice, as pro‐inflammatory factors such as IL‐1α, IL‐1β, IL‐6, and TNF‐α have been shown to act directly on osteoclast precursors to increase osteoclast differentiation,^[^ ^31^ ^]^ and our results showed that treatment with the culture supernatant from the NPY‐ or NPY‐abundant OCY^DMM + Ad libitum^‐CM‐treated monocytes/macrophages could dramatically augment osteoclast formation. Thus, osteocytes can function through NPY to communicate with monocytes/macrophages, which directly results in inflammatory response activation and then indirectly causes subchondral osteoclast overproduction. The detailed molecular mechanism still requires future determination. In addition to the pro‐inflammatory and ‐osteoclastic effects, NPY can also promote the production of various cartilage matrix degradation‐related molecules in chondrocytes,^[^ ^18^ ^]^ which may be another important contributing factor to the positive effect of osteocyte NPY on cartilage degeneration and osteoarthritis.

During osteoarthritis, constant nociceptive input from the inflammatory joint triggers both peripheral and central sensitization and leads to widespread pain.^[^ ^23^, ^32^ ^]^ NPY is a crucial component in nociceptive signaling regulation and exerts a pro‐nociceptive action in the periphery but has an anti‐nociceptive and ‐neuropathic pain effect in the central nervous system.^[^ ^33^ ^]^ Our evidence in present and previous studies showed that brain NPY was not notably affected after DMM and decreased with aging, whereas osteocyte NPY in the bone was markedly increased in both DMM and aging conditions.^[^ ^13^ ^]^ Further results revealed that osteocyte NPY depletion caused significant relief of osteoarthritis pain and reduction of CGRP‐positive sensory nerve fibers in the subchondral bone marrow. These results, together with our evidence showing the direct pro‐neurite outgrowth effect of NPY and OCY^DMM+Ad libitum^‐CM‐CM on neurons, suggest that NPY produced mainly by osteocytes, but not by cells in the brain, interacts with sensory neurons to induce pain hypersensitivity during osteoarthritis.

DMP1 is not exclusively expressed in osteocytes. This protein is also expressed by osteocyte precursors (preosteocytes or late osteoblasts) in the bone as well as by certain cell types in other nonbone tissues such as the brain, tooth germ, pancreas, and kidney.^[^ ^12^, ^34^, ^35^, ^36^, ^37^, ^38^ ^]^ Our previous study has demonstrated that NPY expression in the osteoblasts and in the brain is not markedly influenced in *Dmp1‐iCre*; *Npy^fl/fl^* mice compared with *Npy^fl/fl^* mice.^[^ ^13^ ^]^ *Dmp1‐iCre* does not likely notably affect NPY expression in tooth germ, pancreas, and kidney because NPY is rarely detected in the DMP1‐expressing cells of these tissues.^[^ ^13^ ^]^ Nevertheless, it will be better to carefully assess the expression of *Dmp1‐iCre* in different tissues and precisely check the depletion pattern of NPY in *Dmp1‐iCre*; *Npy^fl/fl^* mice, in order to rule out the contribution of NPY in other cells/tissues to our observed phenotypes in this study, which still needs future determination.

Despite intensive efforts for many years, satisfactory therapies are lacking to halt the progression of osteoarthritis or provide effective and long‐term symptomatic relief for this disease, which finally needs joint replacement with an artificial prosthesis. Results in our study revealed that osteocyte NPY was a multiple pathological processes‐involving molecule during osteoarthritis and could serve as a therapeutic target for osteoarthritis. More importantly, we found that intermittent fasting, a simple, nonpharmacological, nonoperative, painless, and cost‐free dietary intervention approach, could block excess osteocyte NPY production and potently mitigate osteoarthritic phenotypes, including physical activity impairment, pain hypersensitivity, articular cartilage degeneration, joint inflammation, aberrant subchondral bone remodeling, and augmentation of sensory innervation. The antiosteoarthritic effects of intermittent fasting in mice seemed not to be associated with the alteration of body weight, because although intermittent fasting led to marked body weight reduction on fasting days, this change was entirely reversed after subsequent refeeding due to much higher food consumption on refeeding days. However, in humans, overeating on refeeding days can be avoided. Thus, weight reduction following intermittent fasting may also assist in controlling osteoarthritis progression in humans, especially in those with obesity.^[^ ^39^ ^]^

Dysregulation of the gut microbiota has been identified as an important etiological factor in the development of osteoarthritis.^[^ ^40^, ^41^ ^]^ Intermittent fasting can induce various therapeutic benefits on health, such as generating healthy gut microbiota and thus enhancing host function.^[^ ^42^, ^43^ ^]^ Thus, intermittent fasting may act through the regulation of gut microbiota to protect osteoarthritic joints. Nevertheless, if exists, the gut microbiota may finally target osteocyte NPY to mediate the beneficial effects of intermittent fasting against osteoarthritis, because we found that intermittent fasting entirely failed to induce any antiosteoarthritic changes in the osteocyte NPY‐lacking mice subjected to DMM. In our previous studies, we found that extracellular vesicles secreted by the gut‐beneficial bacteria can be transported to the host bone cells and participate in the regulation of bone metabolism,^[^ ^44^, ^45^ ^]^ indicating that the gut microbiota can regulate the function of host bone cells through its secretory factors. Thus, there is a possibility that the gut microbiota is involved in the regulation of osteocyte NPY by intermittent fasting and subsequent improvement of osteoarthritic joints. This still warrants future investigation. In our previous study, we found that NPY expression in osteocytes is increased with aging and osteoporosis, and this alteration occurs due to the overactivity of the sympathetic nervous system (SNS) and the decreased activity of the parasympathetic nervous system (PSNS).^[^ ^13^ ^]^ Considering that fasting can inhibit SNS activity in various peripheral tissues,^[^ ^46^ ^]^ and SNS activation is involved in promoting osteoarthritis progression,^[^ ^47^ ^]^ we hypothesized that the increase of SNS activity may also contribute to the development of DMM‐induced osteoarthritis and the suppression of SNS activity may be an important mechanism by which intermittent fasting down‐regulates osteocyte NPY production and thereby relieve osteoarthritis, all of which still require future determination.

## 4 Experimental Section

### Mice and Treatments

This study received approval from the Ethics Committee at Xiangya Hospital of Central South University (No. 2018081043). C57BL/6 wild‐type (WT) mice, *Dmp1‐iCre* mice, *Npy^fl/fl^* mice, and *Dmp1‐iCre*; *Npy^fl/fl^* mice were used in this study. Since male mice generally exhibit more serious osteoarthritic phenotypes than female mice following DMM operation,^[^ ^48^, ^49^ ^]^ we chose male mice, but not female mice, for all experiments. 3‐ or 16‐month‐old male WT mice were obtained from Hunan SJA Laboratory Animal Co., Ltd (Changsha, China). The source, strategies for the construction of *Dmp1‐iCre* mice and *Npy^fl/−^* mice, and method for mouse genotyping were detailed in our previous study.^[^ ^13^ ^]^ For in vivo experiments, 3‐month‐old male *Dmp1‐iCre*; *Npy^fl/fl^* mice and their control *Npy^fl/fl^* mice were used. The primers used for genotyping were shown as follows: *loxP Npy* allele forward (F): 5′‐TCCCAGACGCCAGTGAACTTGC‐3′, and *loxP Npy* allele reverse (R): 5′‐AGGTGTTCCCAGGTTCTTCTCCC‐3′; *Dmp1*‐*iCre*‐FRT‐F: 5′‐CACGTCCTCTCACTTCTCA CG‐3′, and *Dmp1*‐*iCre*‐FRT‐R: 5′‐CTTTGACAGTGTCTTATCCAATAGCC‐3′. To evaluate the therapeutic effects of intermittent fasting against DMM‐induced osteoarthritis, 3‐month‐old male WT mice were subjected to sham or DMM surgery in the right knees of mice as previously described.^[^ ^50^ ^]^ One week later, the sham or DMM mice were fed ad libitum with a standard diet or received 2, 4, 8, or 12 weeks (corresponding to one, two, four, or six cycles) of intermittent fasting (one cycle: 24 h of daily intermittent fasting for a week and refeeding/nonfasting daily for another week). On the fasting days, the mice were allowed to drink ad libitum but were deprived of food. On the refeeding days, the mice had ad libitum access to both water and food. The body weight and food consumption of these mice were recorded during a 2‐week‐long observation period. To assess the benefits of intermittent fasting in aging‐induced osteoarthritic mice, 16‐month‐old male WT mice were subjected to either ad libitum feeding or intermittent fasting for 8 weeks (four cycles). To examine the involvement of osteocyte NPY in DMM‐induced osteoarthritis, 3‐month‐old male *Dmp1‐iCre*; *Npy^fl/fl^* mice, and *Npy^fl/fl^* mice underwent sham or DMM operation, followed by receiving ad libitum feeding or intermittent fasting for 4 or 8 weeks. Once finished at the indicated time points, behavioral tests were conducted on these mice. After that, the mice were killed to harvest the knee joints and brain tissues for the following analyses. The researchers performing behavioral tests, µCT scanning, and µCT analysis were blinded to group assignment during experiments. Then, the data were checked by the researchers responsible for statistical analysis and Figure preparation, who were not blinded to group assignment.

### Footprint Test

To evaluate the locomotor function and motor coordination of mice by footprint test, a straight narrow tunnel (10 cm wide, 10 cm height, and 60 cm long) was prepared and a sheet of white paper was placed on the bottom of the tunnel for recording the footprints. The paws of the tested mice were dipped in nontoxic waterproof paint (forepaws: red paint; hind paws: black paint) and then allowed to walk freely along the tunnel, where the mouse footprints would be left behind on the white paper. The forelimb and hindlimb stride lengths were measured.

### Motor Activity Tracking

The tested mice were placed in the cages and their locomotor activities were monitored with an action camera for 1 h. The locomotor behavior was analyzed using a multi‐rodent tracking system^[^ ^51^ ^]^ and the motion trajectory diagram was recorded to measure the total distance of movement.

### Rotarod Test

To assess the motor coordination and balance of mice by rotarod test, the mice were placed on a rotating rotarod (Unibiolab, Beijing, China) with a diameter of 3 cm and allowed to acclimate to the testing environment for 1 min. The rotation was started at a slow speed and gradually increased to 60 rpm at an accelerated speed of 3 rpm. Three training sessions per day were conducted for three consecutive days, followed by test sessions to record and compare the latency of the mice to fall off the rotarod.

### Balance Beam Test

The motor coordination and balance of mice were also examined by the balance beam test. Briefly, a horizontal wooden beam (0.9 cm wide, 0.9 cm height, and 20 cm long) with a dark box at the end of the beam was prepared and positioned above two platforms with a height of 40 cm. The tested mice were placed on the beam and allowed to traverse the beam to reach the dark box. After three training sessions a day for 3 consecutive days, test trials were conducted and repeated three times per day. The time taken to complete this task was recorded and the speed of crossing the beam was measured.

### Pain Sensitivity Test

Von Frey's paw withdrawal threshold test was performed to detect the pain behavior of mice. A series of calibrated Von Frey filaments (North Coast Medical, Inc. Morgan Hill, CA, USA) with varying bending forces (0.04–2.0 g) were sequentially applied to the plantar surface of the mouse hind paws. The nocifensive responses of mice were recorded upon filament application, such as paw withdrawal, flinching, licking, or jumping. If paw withdrawal responses were observed, the next filament with a lower bending force was used. The minimal force filament for which the mice displayed paw withdrawal responses at least six times in ten stimulations was recorded as the Von Frey paw withdrawal threshold. The test was repeated three times with a 20 min interval.

### µCT Analysis

After removal of the soft tissues, the knee joints were obtained and fixed with 4% paraformaldehyde (PFA) for 2 days. Then, the samples were analyzed using µCT (vivaCT80; SCANCO Medical AG, Bruettisellen, Switzerland). The scanning parameters including X‐ray voltage, current, integration time, and resolution were set to 70 kv, 400 µA, 400 ms, and 11.4 µm per pixel, respectively. The µCT images of the tibial subchondral bones were reconstructed, analyzed, and visualized by a software called NRecon, CTAn v1.11, and µCTVol v2.0, respectively. The whole subchondral bone medial compartment was the region of interest (ROI) for analyzing subchondral Tb. BV/TV, SBP. Th, and Tb. Pf.

### Histological Staining

The knee joints were fixed in 4% PFA for 2 days and decalcified in 0.5% EDTA for a week. After dehydration and transparency, the joint samples were embedded in paraffin, cut into 5‐µm‐thick sections, and processed for safranin O‐fast green staining with reagents purchased from Servicebio (Cat. No. G1371; Wuhan, China). The cartilage glycosaminoglycans and proteoglycan were stained by safranin O (red color), while the noncartilaginous tissues were stained by fast green. The stained sections were observed with an optical microscope and the mean staining intensity for safranin O was measured using the Image‐Pro Plus 6 software. The histological changes of the tibial cartilages were also detected by HE staining using reagents from Servicebio (Cat. No. G1005). OARSI scores were evaluated based on the safranin O‐fast green and HE staining images using a 0–6 subjective scoring system as described previously,^[^ ^52^ ^]^ in order to assess the extent of osteoarthritic damage. Osteoclast formation in the subchondral bone was evaluated by TRAP staining using a commercial kit (Cat. No. 387A; Sigma‐Aldrich, St. Louis, MO, USA). TRAP‐positive osteoclasts were counted and the number of osteoclasts per bone surface (N. OCs/BS/mm) was measured with the Image‐Pro Plus 6 software.

### Immunostaining

For immunohistochemical staining, the paraffin‐embedded knee joint and brain tissues were prepared and sectioned into 5‐µm‐thick slices, followed by deparaffinization, rehydration, and antigen retrieval. The sections were blocked by 3% hydrogen peroxide and subsequently by 10% normal goat serum (Cat. No. SL038; Solarbio, Beijing, China), followed by incubation with the primary antibodies and then the secondary antibodies. After washing, the sections were detected under an optical microscope, and the images were obtained. For immunofluorescence staining, the knee joint sections were heated in Tris‐EDTA buffer for antigen retrieval, treated with spontaneous fluorescence quenching agent (Cat. No. G1221; Servicebio), blocked with 10% normal goat serum, and then incubated with primary antibodies. After washing, the sections were incubated with the secondary antibodies, stained with DAPI (Cat. No. H‐1200‐10; Vector Laboratories, Burlingame, USA), and photographed under a fluorescence microscope (Carl Zeiss ApoTome, Jena, Germany). The mean intensities for the positively stained areas were measured by Image‐Pro Plus 6 software. Anti‐IL‐6 (Cat. No. 66146‐1‐lg) and anti‐TNF‐α (Cat. No. 17590‐1‐AP) were purchased from ProteinTech (Chicago, USA). Anti‐COL2A1 (Cat. No. GB11021) and antiaggrecan (Cat. No. GB11373) were obtained from Servicebio. Anti‐CGRP (Cat. No. ab36001) and anti‐NPY (Cat. No. 11976) were bought from Abcam (Cambridge, Britain) and Cell Signaling Technology (Danvers, USA), respectively.

### Cell Culture

The procedures for the isolation of primary osteocytes were described in detail in our previous study.^[^ ^13^ ^]^ Briefly, the femurs and tibias were collected, cut into bone pieces, and then subjected to digestion using collagenase type IA (300 U mL^−1^; Cat. No. C9891; Sigma‐Aldrich) three times. After washing, the bone pieces were alternately treated with EDTA (5 mm in magnesium‐and calcium‐free Dulbecco's PBS containing 1% bovine serum albumin) and collagenase type IA three times. The osteocyte‐enriched fractions along with the resulting bone particles were obtained and seeded onto the collagen‐coated culture plates with α‐MEM containing 5% fetal bovine serum (FBS; Cat. No. 10091–148; Gibco, Grand Island, USA), 5% fetal calf serum (FCS; Cat. No. 16010–159; Gibco), and 1% Penicillin‐Streptomycin (PS; Cat. No. P7630; Solarbio, Beijing, China). The monocytes/macrophages RAW264.7 cells were obtained from ATCC (Rockville, USA) and cultured in high glucose Dulbecco's Modified Eagle Medium (DMEM; Cat. No. 11965‐092; Gibco) containing 10% FBS and 1% PS. The neuronal cells CAD were cultured in DMEM/Nutrient Mixture F‐12 (DMEM/F‐12; Cat. No. 11330‐032; Gibco) with 10% FBS and 1% PS. Cells grow at 37 °C in 5% CO_2_.

### OCY‐CM and MM‐CM Preparation

OCY‐CM was prepared from the culture supernatants of osteocytes from mice in Sham + Ad libitum, DMM + Ad libitum, and DMM + Fasting groups. For OCY‐CM preparation, two mice‐derived femurs and tibias were pooled together and processed for osteocyte isolation. In total, six mice per group were used and three osteocyte samples were obtained for OCY‐CM preparation in each group. For MM‐CM preparation, the culture of RAW264.7 cells was added with OCY‐CM from different groups, NPY (0.1 nm; Cat. No. HY‐P0198A; MedChemExpress, Monmouth Junction, USA) + un‐cultured control medium of osteocytes (NPY group for short), or vehicle of NPY + un‐cultured control medium of osteocytes (Control group for short). After treatment for 24 h, the culture medium of RAW264.7 cells in different groups was changed to a fresh complete culture medium without adding the substances described above. After culture for another 24 h, MM‐CM from different groups (culture medium of RAW264.7 cells in OCY^Sham + Ad libitum^‐CM, OCY^DMM + Ad libitum^‐CM, OCY^DMM + Fasting^‐CM, NPY, and Control treatment groups) was collected. The culture supernatants were centrifugated at 300×g for 10 min, followed by another centrifugation at 2000×g for 30 min to eliminate dead cells and debris. The obtained supernatants were transferred to an Amicon Ultra‐4 Centrifugal Filter Unit (3 kDa; Millipore, Billerica, USA) and centrifugated at 4000×g for concentration to 100–200 µL. The concentrated supernatants were then subjected to protein content analysis using a kit from Thermo Fisher Scientific (Cat. No. 23227; Madison, Waltham, USA). For the following experiments, OCY‐CM and MM‐CM were used at the dose of 300 µg mL^−1^.

### ELISA

The concentration of NPY in the control medium, OCY^Sham+Ad libitum^‐CM, OCY^DMM+Ad libitum^‐CM, and OCY^DMM+Fasting^‐CM was tested using ELISA kit from Elabscience (Cat. No. E‐EL‐M0820; Wuhan, China). The procedures were conducted following the instructions provided by the kit protocol.

### qRT‐PCR

Total RNA was extracted from RAW264.7 cells treated with control medium, OCY^Sham + Ad libitum^‐CM, OCY^DMM + Ad libitum^‐CM, or OCY^DMM + Fasting^‐CM, and subjected to cDNA synthesis with a commercial kit (Cat. No. E047‐01B; Novoprotein Scientific Inc, Shanghai, China), followed by qRT‐PCR analysis of the indicated gene expression with reagents from Selleckchem (Cat. No. B24202; Houston, USA) and an analysis system from Funglyn Biotech Inc (FTC‐3000; Toronto, Canada). The *Gapdh* gene served as the internal control. Primers were as follows: *Il‐1α*‐F, 5′‐CGAAGACTACAGTTCTGCCATT‐3′, and *Il‐1α*‐R, 5′‐GACGTTTCAGAGGTTCTCAGAG‐3′; *Il‐1β*‐F, 5′‐GAAATGCCACCTTTTGACAGTG‐3′, and *Il‐1β*‐R, 5′‐TGGATGCTCTCATCAGGACAG‐3′; *Il‐6*‐F, 5′‐TAGTCCTTCCTACCCCAATTTCC‐3′, and *Il‐6*‐R, 5′‐TTGGTCCTTAGCCACTCCTTC‐3′; *Tnf‐α*‐F, 5′‐TGAACTTCGGGGTGATCGGTC‐3′, and *Tnf‐α*‐R, 5′‐CACTTGGTGGTTTGCTACGACG‐3′; *Gapdh*‐F, 5′‐CACCATGGAGAAGGCCGGGG‐3′, and *Gapdh*‐R, 5′‐GACGGACACATTGGGGGTAG‐3′.

### Western Blotting

Protein lysates from RAW264.7 cells with different treatments were prepared and separated by SDS‐PAGE. After being transferred onto PVDF membranes (0.2 µm; Cat. No. ISEQ00010; Millipore, MA, USA), the membranes were blocked with 5% skim milk for 30 min and incubated with anti‐IL‐1α (1:3000; Cat. No. 16765‐1‐AP; ProteinTech, Chicago, USA), anti‐IL‐1β (1:2000; Cat. No. GB115602; Servicebio, Wuhan, China), anti‐IL‐6 (1:3000; Cat. No. 21865‐1‐AP; ProteinTech), anti‐TNF‐α (1:2000; Cat. No. GB11188; Servicebio), or anti‐GAPDH (1:2000; Cat. No. GB11188; Servicebio) at 4 °C overnight. After that, the membranes were washed and incubated with secondary antibodies (Cat. No. 3678S; Cell Signaling Technology) for 60 min at room temperature, followed by band visualization using an Advansta ECL detection kit (Cat. No. K‐12045‐D50; San Jose, USA).

### Neurite Growth Assay

To induce the growth of neurites in neuronal cell line CAD, the complete culture medium of CAD was changed to serum‐free DMEM/F‐12 added with NPY (0.1 nm), OCY^Sham+Ad libitum^‐CM, OCY^DMM+Ad libitum^‐CM, OCY^DMM + Fasting^‐CM, or an equal volume of un‐cultured medium/vehicle (control). After incubation for 3 days, the cells were stained with Calcein‐AM (2 µm; Cat. No. 40747ES76; Yeasen Biotech, Shanghai, China) and detected under a microscope. The images were obtained to assess the neurite lengths.

### Osteoclastic Differentiation Assay

RAW264.7 cells were plated in a 48‐well plate (1 × 10^4^ cells per well) and incubated in osteoclast differentiation medium containing RANKL (100 ng mL^−1^; Cat. No. 315‐11; PeproTech, Rocky Hill, USA) and supplemented with vehicle, NPY (0.1 nm), OCY^Sham + Ad libitum^‐CM, OCY^DMM + Ad libitum^‐CM, OCY^DMM + Fasting^‐CM, or MM‐CM from different treatment groups. The induction medium was refreshed every other day throughout an 8‐day differentiation period. Then, the cells were processed for TRAP staining with a commercial kit (Cat. No. 387A; Sigma‐Aldrich) and the positively stained osteoclasts (>3 nuclei) in each well were counted under a microscope. The images of stained cells were photographed at 100× magnification.

### Statistical Analysis

The software GraphPad Prism 8 was used for statistical analyses of all data (mean ± SD). Unpaired, two‐tailed Student's *t*‐test and one‐ or two‐way analysis of variance (ANOVA) combined with Bonferroni *post hoc* test were performed for two and multiple‐group comparisons, respectively. Statistical significance was considered when *p*‐value <0.05.

## Conflict of Interest

The authors declare no conflict of interest.

## Author Contributions

Y.‐X.Q. and S.‐S.R. contributed equally to this work. C.‐Y.C. and H.X. designed this study and wrote the manuscript. Y.‐X.Q., S.‐S.R., Y.‐J.T., Z.W., H.Y., T.‐F.W., Z.‐H.H., X.W., C.‐G.H., H.‐J.Z., Y.L., Y.X.D. H.Z., L.Z., and B.B.L. performed the experiments or/and analyzed the data. Y.‐X.Q., S.‐S.R., C.‐Y.C., and Z.W. prepared the figures. X.‐Y.H., Y.Z., Z.‐X.W., and W.D. provided technical support.

[Correction added on July 12, 2024, after first online publication: Equal contribution statement is added in Author Contributions section.]

## Supporting information

Supporting Information

## Data Availability

The data that support the findings of this study are available from the corresponding author upon reasonable request.

## References

[1] L. Yue , J. Berman , JAMA, J. Am. Med. Assoc. 2022, 327, 1300. 10.1001/jama.2022.1980 35380583

[2] Q. Yao , X. Wu , C. Tao , W. Gong , M. Chen , M. Qu , Y. Zhong , T. He , S. Chen , G. Xiao , Signal Transduct. Target Ther. 2023, 8, 56. 36737426 10.1038/s41392-023-01330-w PMC9898571

[3] W. Jiang , Y. Jin , S. Zhang , Y. Ding , K. Huo , J. Yang , L. Zhao , B. Nian , T. P. Zhong , W. Lu , H. Zhang , X. Cao , K. M. Shah , N. Wang , M. Liu , J. Luo , Bone Res. 2022, 10, 27. 35260562 10.1038/s41413-022-00201-4 PMC8904489

[4] K. A. Varady , S. Cienfuegos , M. Ezpeleta , K. Gabel , Nat. Rev. Endocrinol. 2022, 18, 309. 35194176 10.1038/s41574-022-00638-x

[5] E. Serger , L. Luengo‐Gutierrez , J. S. Chadwick , G. Kong , L. Zhou , G. Crawford , M. C. Danzi , A. Myridakis , A. Brandis , A. T. Bello , F. Müller , A. Sanchez‐Vassopoulos , F. De Virgiliis , P. Liddell , M. E. Dumas , J. Strid , S. Mani , D. Dodd , S. Di Giovanni , Nature 2022, 607, 585. 35732737 10.1038/s41586-022-04884-x

[6] I. Minciună , S. Gallage , M. Heikenwalder , S. Zelber‐Sagi , J. F. Dufour , Hepatology 2023, 78, 1290. 37057877 10.1097/HEP.0000000000000330

[7] M. J. Luo , S. S. Rao , Y. J. Tan , H. Yin , X. K. Hu , Y. Zhang , Y. W. Liu , T. Yue , L. J. Chen , L. Li , Y. R. Huang , Y. X. Qian , Z. Z. Liu , J. Cao , Z. X. Wang , Z. W. Luo , Y. Y. Wang , K. Xia , S. Y. Tang , C. Y. Chen , H. Xie , Theranostics 2020, 10, 3779. 32206122 10.7150/thno.44115 PMC7069085

[8] S. Schmidt , R. Stange , E. Lischka , M. Kiehntopf , T. Deufel , D. Loth , C. Uhlemann , Forsch. Komplementmed. 2010, 17, 87. 20484916 10.1159/000285479

[9] S. Drinda , S. Franke , S. Schmidt , K. Stoy , T. Lehmann , G. Wolf , T. Neumann , Complement. Med. Res. 2018, 25, 167. 29433120 10.1159/000486237

[10] S. Park , B. K. Shin , Br. J. Nutr. 2022, 127, 55. 33750486 10.1017/S0007114521000829

[11] Y. Y. Qin , X. R. Huang , J. Zhang , W. Wu , J. Chen , S. Wan , X. Y. Yu , H. Y. Lan , Mol. Ther. 2022, 30, 881. 34628054 10.1016/j.ymthe.2021.10.005 PMC8821956

[12] J. C. Igwe , X. Jiang , F. Paic , L. Ma , D. J. Adams , P. A. Baldock , C. C. Pilbeam , I. Kalajzic , J. Cell Biochem. 2009, 108, 621. 19670271 10.1002/jcb.22294 PMC2754602

[13] Y. Zhang , C. Y. Chen , Y. W. Liu , S. S. Rao , Y. J. Tan , Y. X. Qian , K. Xia , J. Huang , X. X. Liu , C. G. Hong , H. Yin , J. Cao , S. K. Feng , Z. H. He , Y. Y. Li , Z. W. Luo , B. Wu , Z. Q. Yan , T. H. Chen , M. L. Chen , Y. Y. Wang , Z. X. Wang , Z. Z. Liu , M. J. Luo , X. K. Hu , L. Jin , T. F. Wan , T. Yue , S. Y. Tang , H. Xie , Adv. Sci. 2021, 8, e2100808. 10.1002/advs.202100808 PMC8693044 34719888

[14] C. Park , J. Kim , S. B. Ko , Y. K. Choi , H. Jeong , H. Woo , H. Kang , I. Bang , S. A. Kim , T. Y. Yoon , C. Seok , W. Im , H. J. Choi , Nat. Commun. 2022, 13, 853. 35165283 10.1038/s41467-022-28510-6 PMC8844075

[15] N. Horio , S. D. Liberles , Nature 2021, 592, 262. 33658716 10.1038/s41586-021-03299-4 PMC8035273

[16] W. C. Chen , Y. B. Liu , W. F. Liu , Y. Y. Zhou , H. F. He , S. Lin , Front. Immunol. 2020, 11, 580378. 33123166 10.3389/fimmu.2020.580378 PMC7573154

[17] L. Wang , L. Zhang , H. Pan , S. Peng , M. Lv , W. W. Lu , BMC Musculoskelet. Disord. 2014, 15, 319. 25262001 10.1186/1471-2474-15-319 PMC4195915

[18] X. Kang , Z. Qian , J. Liu , D. Feng , H. Li , Z. Zhang , X. Jin , Z. Ma , M. Xu , F. Li , Y. Zhang , X. Gao , H. Sun , S. Wu , J. Bone Miner. Res. 2020, 35, 1375. 32101625 10.1002/jbmr.3991

[19] W. Hu , Y. Chen , C. Dou , S. Dong , Ann. Rheum. Dis. 2021, 80, 413. 33158879 10.1136/annrheumdis-2020-218089 PMC7958096

[20] M. Xiao , W. Zhang , W. Liu , L. Mao , J. Yang , L. Hu , S. Zhang , Y. Zheng , A. Liu , Q. Song , Y. Li , G. Xiao , Z. Zou , X. Bai , Blood 2021, 137, 3533. 33684929 10.1182/blood.2020007731 PMC8225922

[21] W. Zhou , M. Murakami , S. Hasegawa , F. Yoshizawa , K. Sugahara , Comp. Biochem. Physiol. A. Mol. Integr. Physiol. 2005, 141, 146. 15982913 10.1016/j.cbpb.2005.04.015

[22] S. Kassab , T. Abdul‐Ghaffar , D. S. Nagalla , U. Sachdeva , U. Nayar , Ann. Saudi. Med. 2004, 24, 345. 15573845 10.5144/0256-4947.2004.345 PMC6148150

[23] S. Zhu , J. Zhu , G. Zhen , Y. Hu , S. An , Y. Li , Q. Zheng , Z. Chen , Y. Yang , M. Wan , R. L. Skolasky , Y. Cao , T. Wu , B. Gao , M. Yang , M. Gao , J. Kuliwaba , S. Ni , L. Wang , C. Wu , D. Findlay , H. K. Eltzschig , H. W. Ouyang , J. Crane , F. Q. Zhou , Y. Guan , X. Dong , X. Cao , J. Clin. Invest. 2019, 129, 1076. 30530994 10.1172/JCI121561 PMC6391093

[24] M. L. Ji , H. Jiang , Z. Li , R. Geng , J. Z. Hu , Y. C. Lin , J. Lu , Nat. Commun. 2022, 13, 7658. 36496445 10.1038/s41467-022-35424-w PMC9741608

[25] S. S. Rao , Y. Hu , P. L. Xie , J. Cao , Z. X. Wang , J. H. Liu , H. Yin , J. Huang , Y. J. Tan , J. Luo , M. J. Luo , S. Y. Tang , T. H. Chen , L. Q. Yuan , E. Y. Liao , R. Xu , Z. Z. Liu , C. Y. Chen , H. Xie , Bone Res. 2018, 6, 9. 29619269 10.1038/s41413-018-0012-0 PMC5876344

[26] Y. Qi , J. K. Wang , M. McMillian , D. M. Chikaraishi , J. Neurosci. 1997, 17, 1217. 9006967 10.1523/JNEUROSCI.17-04-01217.1997 PMC6793738

[27] Y. Hu , X. Chen , S. Wang , Y. Jing , J. Su , Bone Res. 2021, 9, 20. 33731688 10.1038/s41413-021-00147-z PMC7969608

[28] L. Zhang , C. Wen , Int. J. Mol. Sci. 2021, 22, 6522. 34204587 10.3390/ijms22126522 PMC8233862

[29] G. Dai , H. Xiao , J. Liao , N. Zhou , C. Zhao , W. Xu , W. Xu , X. Liang , W. Huang , Int. J. Mol. Med. 2020, 46, 167. 32319543 10.3892/ijmm.2020.4576 PMC7255453

[30] S. Park , T. Komatsu , H. Hayashi , R. Mori , I. Shimokawa , Biomedicines 2021, 9, 1739. 34829968 10.3390/biomedicines9111739 PMC8615496

[31] N. Komatsu , H. Takayanagi , Nat. Rev. Rheumatol. 2022, 18, 415. 35705856 10.1038/s41584-022-00793-5

[32] P. G. Conaghan , A. D. Cook , J. A. Hamilton , P. P. Tak , Nat. Rev. Rheumatol. 2019, 15, 355. 31068673 10.1038/s41584-019-0221-y

[33] M. Diaz‐delCastillo , D. P. D. Woldbye , A. M. Heegaard , Neuroscience 2018, 387, 162. 28890052 10.1016/j.neuroscience.2017.08.050

[34] I. Kalajzic , A. Braut , D. Guo , X. Jiang , M. S. Kronenberg , M. Mina , M. A. Harris , S. E. Harris , D. W. Rowe , Bone 2004, 35, 74. 15207743 10.1016/j.bone.2004.03.006

[35] S. Toyosawa , S. Shintani , T. Fujiwara , T. Ooshima , A. Sato , N. Ijuhin , T. Komori , J. Bone Miner. Res. 2001, 16, 2017. 11697797 10.1359/jbmr.2001.16.11.2017

[36] N. Kamiya , M. Takagi , Histochem. J. 2001, 33, 545. 12005026 10.1023/a:1014955925339

[37] M. Terasawa , R. Shimokawa , T. Terashima , K. Ohya , Y. Takagi , H. Shimokawa , J. Bone Miner. Metab. 2004, 22, 430. 15316863 10.1007/s00774-004-0504-4

[38] B. Jing , C. Zhang , X. Liu , L. Zhou , J. Liu , Y. Yao , J. Yu , Y. Weng , M. Pan , J. Liu , Z. Wang , Y. Sun , Y. E. Sun , Protein Cell 2018, 9, 298. 28822114 10.1007/s13238-017-0449-8 PMC5829272

[39] S. Babu , A. Vaish , R. Vaishya , A. Agarwal , J. Clin. Orthop. Trauma 2021, 16, 70. 33717941 10.1016/j.jcot.2020.12.020 PMC7920092

[40] Z. Wei , F. Li , G. Pi , Front. Cell Infect. Microbiol. 2022, 12, 812596. 35372125 10.3389/fcimb.2022.812596 PMC8966131

[41] Z. Huang , J. Chen , B. Li , B. Zeng , C. H. Chou , X. Zheng , J. Xie , H. Li , Y. Hao , G. Chen , F. Pei , B. Shen , V. B. Kraus , H. Wei , X. Zhou , L. Cheng , Ann. Rheum. Dis. 2020, 79, 646. 32205337 10.1136/annrheumdis-2019-216471 PMC7384301

[42] F. Cignarella , C. Cantoni , L. Ghezzi , A. Salter , Y. Dorsett , L. Chen , D. Phillips , G. M. Weinstock , L. Fontana , A. H. Cross , Y. Zhou , L. Piccio , Cell Metab. 2018, 27, 1222. 29874567 10.1016/j.cmet.2018.05.006 PMC6460288

[43] M. Barati , A. Ghahremani , H. Namdar Ahmadabad , Autoimmun. Rev. 2023, 22, 103408. 37572827 10.1016/j.autrev.2023.103408

[44] C. Y. Chen , S. S. Rao , T. Yue , Y. J. Tan , H. Yin , L. J. Chen , M. J. Luo , Z. Wang , Y. Y. Wang , C. G. Hong , Y. X. Qian , Z. H. He , J. H. Liu , F. Yang , F. Y. Huang , S. Y. Tang , H. Xie , Sci. Adv. 2022, 8, eabg8335. 35417243 10.1126/sciadv.abg8335 PMC9007505

[45] J. H. Liu , C. Y. Chen , Z. Z. Liu , Z. W. Luo , S. S. Rao , L. Jin , T. F. Wan , T. Yue , Y. J. Tan , H. Yin , F. Yang , F. Y. Huang , J. Guo , Y. Y. Wang , K. Xia , J. Cao , Z. X. Wang , C. G. Hong , M. J. Luo , X. K. Hu , Y. W. Liu , W. Du , J. Luo , Y. Hu , Y. Zhang , J. Huang , H. M. Li , B. Wu , H. M. Liu , T. H. Chen , et al., Adv. Sci. 2021, 8, 2004831. 10.1002/advs.202004831 PMC8097336 33977075

[46] J. B. Young , L. Landsberg , Obes. Res. 1997, 5, 646. 9449153 10.1002/j.1550-8528.1997.tb00590.x

[47] G. Rösch , F. Zaucke , Z. Jenei‐Lanzl , Am. J. Physiol. Cell Physiol. 2023, 325, C365. 37335027 10.1152/ajpcell.00039.2023

[48] H. Fang , F. Beier , Nat. Rev. Rheumatol. 2014, 10, 413. 24662645 10.1038/nrrheum.2014.46

[49] Y. Zhao , B. Liu , C. J. Liu , J. Vis. Exp. 2014, 25, e50924. 10.3791/50924 PMC4131755 24638128

[50] S. S. Glasson , T. J. Blanchet , E. A. Morris , Osteoarthr. Cartil. 2007, 15, 1061. 10.1016/j.joca.2007.03.006 17470400

[51] B. Liu , Y. Qian , J. Wang , BMC Neurosci. 2023, 24, 23. 36973649 10.1186/s12868-023-00787-3 PMC10044788

[52] S. S. Glasson , M. G. Chambers , W. B. Van Den Berg , C. B. Little , Osteoarthr. Cartil. 2010, 18, S17. 10.1016/j.joca.2010.05.025 20864019

