# Supplementary material for: Intermittent Fasting Targets Osteocyte Neuropeptide Y to Relieve Osteoarthritis
Source: Adv Sci (Weinh). 2024 Jul 8;11(34):2400196. doi: 10.1002/advs.202400196 (PMC11425897; doi:10.1002/advs.202400196)
Supplement: Supplementary file 1 — Supporting Information [file ADVS-11-2400196-s001.docx]

**Supplementary Materials for**

**Intermittent fasting targets osteocyte neuropeptide Y to relieve osteoarthritis**

Yu-Xuan Qian^#^, Shan-Shan Rao^#^, Yi-Juan Tan, Zun Wang, Hao Yin, Teng-Fei Wan, Ze-Hui He, Xin Wang, Chun-Gu Hong, Hai-Jin Zeng, Yi Luo, Yan-Xin Duan, Hao Zhu, Xin-Yue Hu, Ling Zou, Yan Zhang, Bing-Bing Liu, Zhen-Xing Wang, Wei Du, Chun-Yuan Chen**^*^**, Hui Xie**^*^**

**^#^** Yu-Xuan Qian and Shan-Shan Rao contributed equally to this work.

^*^Corresponding authors: Hui Xie ([huixie@csu.edu.cn](mailto:huixie@csu.edu.cn)); Chun-Yuan Chen ([chency19@csu.edu.cn](mailto:chency19@csu.edu.cn)).

**This file includes:**

figures S1-4


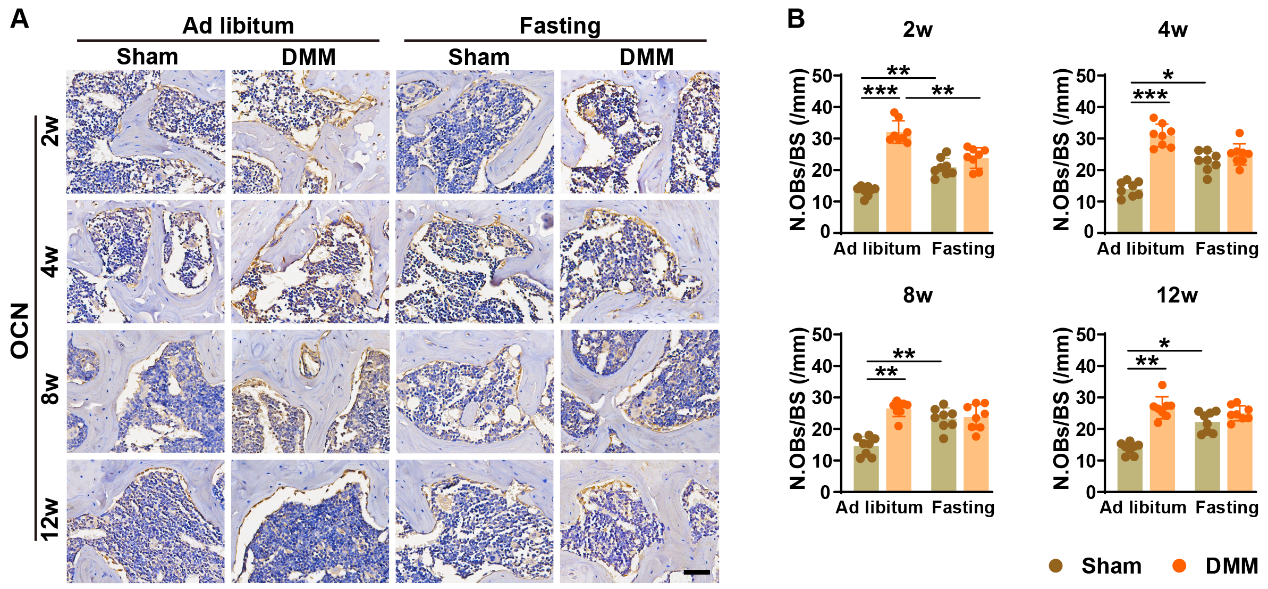


**Figure S1 Intermittent fasting blocks DMM-induced excess osteogenesis. A, B**) Representative OCN staining images (**A**) and quantification of the numbers of the OCN-stained osteoblasts per bone surface (**B**; N. OBs/BS/mm) in the tibial subchondral bones from mice receiving ad libitum feeding or intermittent fasting for 2, 4, 8, or 12 weeks. Scale bars: 100 μm. n = 8 per group. Data are presented as mean ± SD. Two-way ANOVA combined with Bonferroni *post hoc* test. **^*^***P* < 0.05, **^**^***P* < 0.01, **^***^***P* < 0.001.


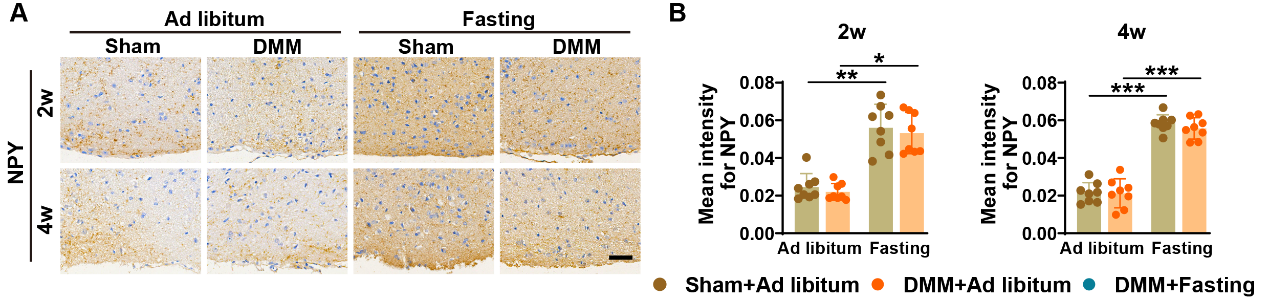


**Figure S2 Effect on intermittent fasting on brain NPY expression in DMM mice.** (**A, B**) Representative immunohistochemical staining images of NPY (**A**) and quantification of the mean staining intensity for NPY (**B**) in the brain tissues from sham- or DMM-operated 3-month-old young mice receiving ad libitum feeding or intermittent fasting for 2 or 4 weeks. Scale bars: 50 μm. n = 8 per group. Data are presented as mean ± SD. Two-way ANOVA combined with Bonferroni *post hoc* test. **^*^***P* < 0.05, **^**^***P* < 0.01, **^***^***P* < 0.001.


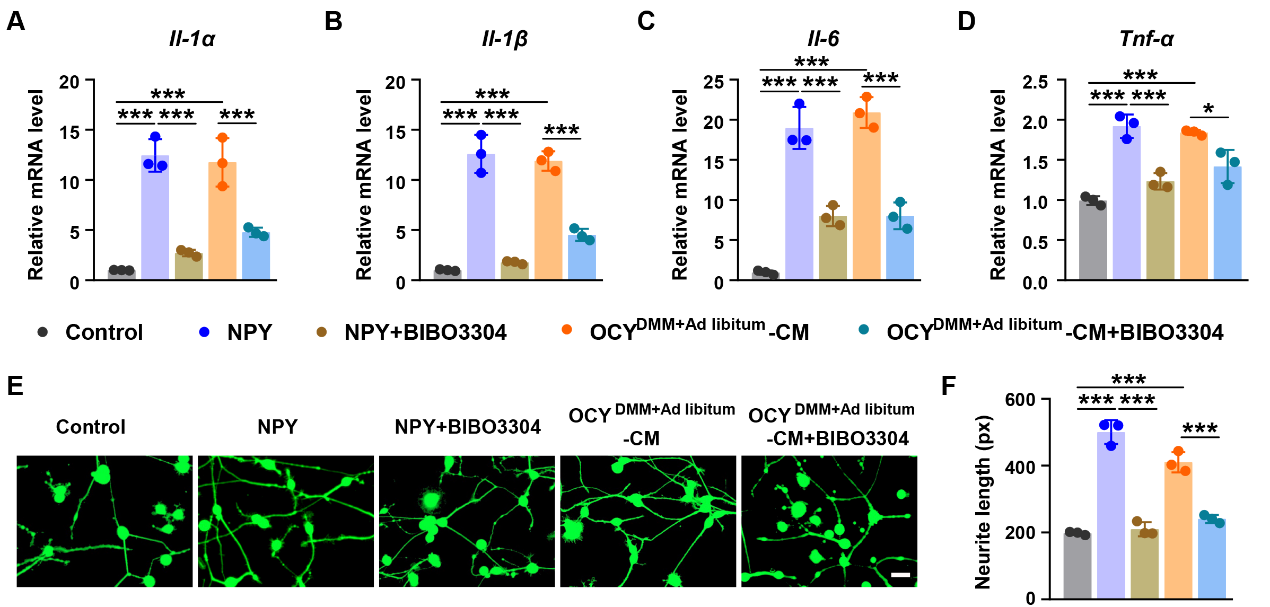


**Figure S3 NPY and OCY^DMM+Ad libitum^-CM function through Y1R to stimulate inflammation and neurite outgrowth.** (**A-D**) qRT-PCR of *Il-1α* (**A**), *Il-1β* (**B**), *Il-6* (**C**), and *Tnf-α* (**D**) in monocyte/macrophage cell line RAW264.7 with different treatments. n = 3 per group. (**E, F**) Representative staining images of calcein AM (**E**) and quantification of the neurite lengths (**F**) in the calcein AM-stained live CAD cells. Scale bar: 50 μm. *n* = 3 per group. Data are presented as mean ± SD. Unpaired, two tailed student's *t*-test (Control *vs* NPY groups; Control *vs* OCY^DMM + Ad libitum^-CM groups; NPY *vs* NPY + BIBO3304 groups; OCY^DMM + Ad libitum^-CM *vs* OCY^DMM + Ad libitum^-CM + BIBO3304 groups). **^*^***P* < 0.05, **^**^***P* < 0.01, **^***^***P* < 0.001.


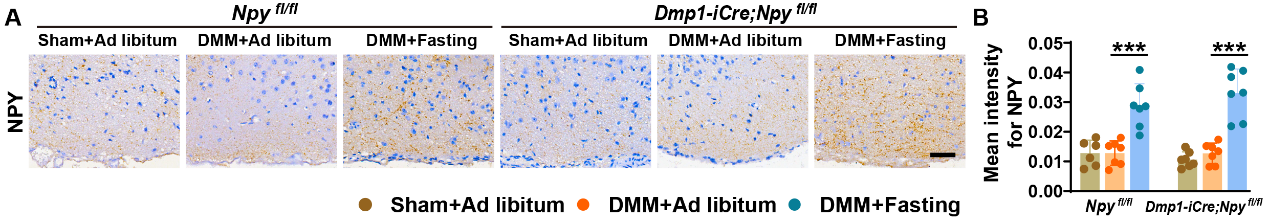


**Figure S4 Brain NPY is not notably affected in *Dmp1-iCre*; *Npy^fl/fl^* mice with or without intermittent fasting.** (**A, B**) Representative immunohistochemical staining images of NPY (**A**) and quantification of the mean staining intensity for NPY (**B**) in the brain tissues from sham- or DMM-operated *Npy^fl/fl^* mice and *Dmp1-iCre*; *Npy^fl/fl^* mice receiving ad libitum feeding or intermittent fasting for 4 weeks. Scale bars: 50 μm. n = 6-7 per group. Data are presented as mean ± SD. Two-way ANOVA combined with Bonferroni *post hoc* test. **^***^***P* < 0.001.
